# Intracytoplasmic sperm injection induces transgenerational abnormalities in mice

**DOI:** 10.1172/JCI170140

**Published:** 2023-11-15

**Authors:** Mito Kanatsu-Shinohara, Yusuke Shiromoto, Narumi Ogonuki, Kimiko Inoue, Satoko Hattori, Kento Miura, Naomi Watanabe, Ayumi Hasegawa, Keiji Mochida, Takuya Yamamoto, Tsuyoshi Miyakawa, Atsuo Ogura, Takashi Shinohara

**Affiliations:** 1 Department of Molecular Genetics, Graduate School of Medicine, Kyoto University, Kyoto, Japan.; 2 AMED-CREST, Chiyodaku, Tokyo, Japan.; 3 Bioresource Engineering Division, RIKEN BioResource Research Center, Ibaraki, Japan.; 4 Division of Systems Medical Science, Center for Medical Science, Fujita Health University, Toyoake, Japan.; 5 Department of Life Science Frontiers, Center for iPS Cell Research and Application, Kyoto University, Kyoto, Japan.; 6 Institute for the Advanced Study of Human Biology (WPI-ASHBi), Kyoto University, Kyoto, Japan.

**Keywords:** Reproductive Biology, Stem cells, Behavior, Fertility

## Abstract

In vitro fertilization (IVF) and intracytoplasmic sperm injection (ICSI) are 2 major assisted reproductive techniques (ARTs) used widely to treat infertility. Recently, spermatogonial transplantation emerged as a new ART to restore fertility to young patients with cancer after cancer therapy. To examine the influence of germ cell manipulation on behavior of offspring, we produced F_1_ offspring by a combination of two ARTs, spermatogonial transplantation and ICSI. When these animals were compared with F_1_ offspring produced by ICSI using fresh wild-type sperm, not only spermatogonial transplantation–ICSI mice but also ICSI-only control mice exhibited behavioral abnormalities, which persisted in the F_2_ generation. Furthermore, although these F_1_ offspring appeared normal, F_2_ offspring produced by IVF using F_1_ sperm and wild-type oocytes showed various types of congenital abnormalities, including anophthalmia, hydrocephalus, and missing limbs. Therefore, ARTs can induce morphological and functional defects in mice, some of which become evident only after germline transmission.

## Graphical abstract

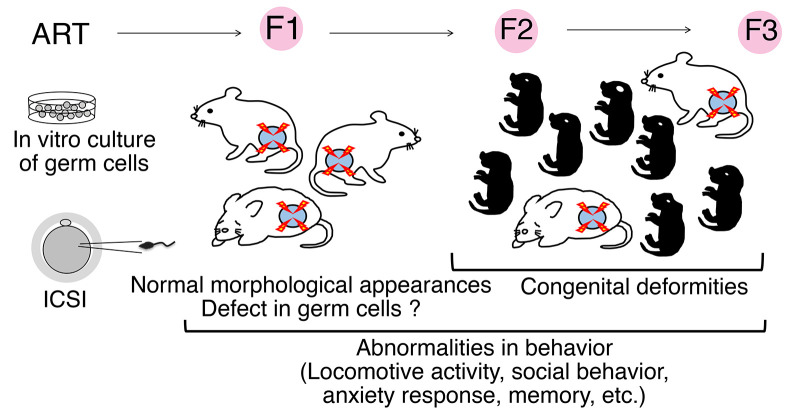

## Introduction

Since its first report in 1978, in vitro fertilization (IVF) has been used in assisted reproductive techniques (ARTs) in humans (1). IVF is often used when a woman’s fallopian tubes are blocked or when a man has a low sperm count. Subsequent development of intracytoplasmic sperm injection (ICSI) greatly expanded the application of ARTs by direct microinjection of a spermatozoon into the oocyte cytoplasm (2). Because ICSI requires only a small number of sperm in testis or epididymis, it is now applied to several types of male factor infertility, including azoospermia and globozoospermia. However, because only 4 animals (2 rabbits and 2 calf) were born as a result of ICSI trials before its clinical application (3), the risk of ICSI has been debated for years (4). Although no association with major congenital abnormalities was found, large epidemiological studies showed increased risks of lower birth weight, minor anomalies, and imprinting disorders (5–10). The risks of impaired cognitive development, neurodevelopmental disorders, and metabolic health have remained inconclusive (11). However, little is known about the effect of ICSI on subsequent generations because of the long human reproductive cycle (12–15). Although one study using mice showed increased apoptosis of spermatocytes, no study showed abnormal phenotype using wild-type sperm (16). More recently, spermatogonial stem cell (SSC) transplantation emerged as a new ART. It is expected to restore fertility in boys who undergo cancer therapy (17). When SSCs are lost owing to cancer treatment in boys before puberty, infertility may be prevented by reintroduction of SSCs after cancer treatment.

Embryonic cells are sensitive to experimental manipulation. For example, in vitro cultures of preimplantation embryos result in “offspring syndrome” in animals, including in cattle and sheep (18). These animals have excessive birth weight, large tongues, umbilical hernia, hypoglycemia, and visceromegaly. These effects result from dysregulation of a set of genes that are expressed only from the maternally or paternally inherited chromosomes, called imprinted genes (19). Cloned animals also exhibit abnormal expression of imprinted genes (20). Proper allelic expression of imprinted genes plays an important role in embryonic and neonatal growth, placental function, and postnatal behavior.

Given these results, it is possible that germ cell manipulation influences offspring health. While the impact of embryo culture on F_1_ offspring has been established and its mechanism is gradually being elucidated (21), few studies have evaluated the effect of SSC transplantation. Here, we evaluated the impact of ARTs using mouse germline stem (GS) cells, which are cultured spermatogonia with enriched SSC activity (22). We initiated this study to examine the effect of spermatogonia transplantation on offspring behavior and produced offspring by ICSI. Analysis of offspring revealed that germ cell manipulation causes transgenerational defects in subsequent generations.

## Results

### Production of F_1_ animals by ICSI.

**Figure 1:**
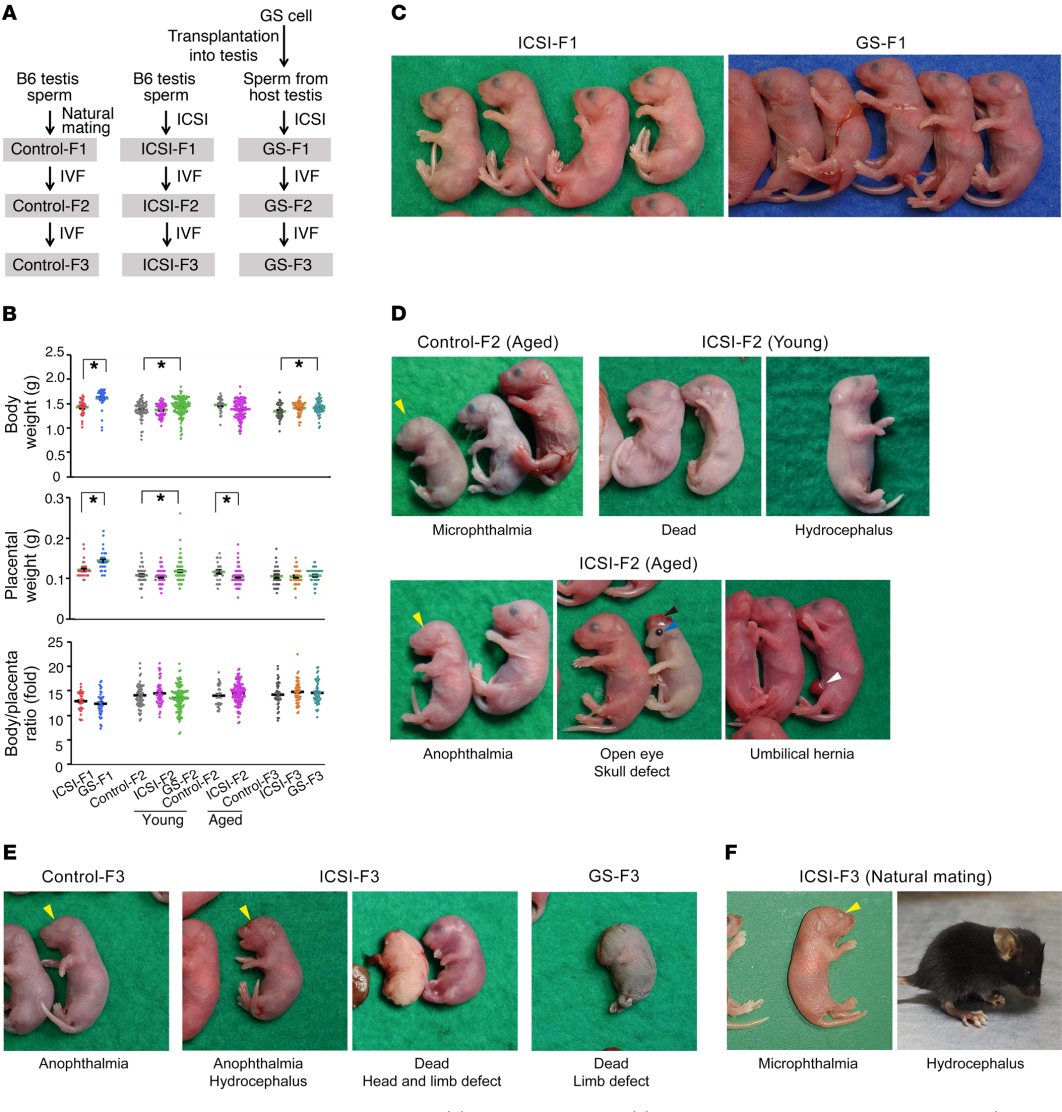
Congenital abnormalities in ICSI-derived offspring. (**A**) Experimental outline. (**B**) Body and placental weight at the time of birth (*n* = 31 for ICSI-F_1_; *n* = 37 for GS-F_1_; *n* = 63 for young control-F_2_; *n* = 50 for young ICSI-F_2_; *n* = 109 for young GS-F_2_; *n* = 25 for aged control-F_2_; *n* = 103 for aged ICSI-F_2_; *n* = 34 for control-F_3_; *n* = 45 for ICSI-F_3_; *n* = 49 for GS-F_3_). (**C**) F_1_ offspring produced by ICSI and SSC transplantation. (**D**) Congenital deformities found in F_2_ offspring produced by IVF using sperm from young (15 months) or aged (25 months) F_1_ mice. (**E**) Congenital deformities found in F_3_ offspring produced by IVF using sperm from F_2_ mice. (**F**) Congenital deformities found in F_3_ offspring produced by natural mating between F_2_ mice. *P < 0.05, 2-tailed Student’s *t* test.

To examine whether SSC manipulation affects ART outcome, we used GS cells from C57BL/6 Tg14(act-EGFP)OsbY01(Green) mice that express *Egfp* gene ubiquitously (B6-GS cells) (Supplemental Figure 1A; supplemental material available online with this article; https://doi.org/10.1172/JCI170140DS1). B6-GS cells appeared very similar to GS cells in a DBA/2 background (DBA-GS cells), which produce offspring by natural mating even after long-term culture (22). Bisulfite sequencing analysis showed typical androgenetic DNA methylation patterns with hypermethylation of *H19* and *Meg3* IG differentially methylated regions (DMRs) and hypomethylation in *Igf2r* and *Snrpn* DMRs in both cell types (Supplemental Figure 1B). Real-time PCR analysis was consistent with the DNA methylation patterns (Supplemental Figure 1C). B6-GS cells were transplanted into the seminiferous tubules of congenitally infertile WBB6F1-W/W^v^ mice (W) to produce sperm (23). Within 3 months, B6-GS cells generated SYCP3^+^ spermatocytes and peanut agglutinin^+^ (PNA^+^) haploid cells (Supplemental Figure 1, D and E). To produce offspring, sperm or elongated spermatids were used for ICSI (24). We also used sperm freshly prepared from green mouse testes as a control (Figure 1A).

After Caesarean section, we found that significantly fewer mice were born from W mice compared with mice born after ICSI using fresh sperm (Supplemental Table 1). The most striking finding was the production of placenta-only offspring (5.2% vs. 0.4%). Bodies and placentas from GS cell–derived mice were larger than those of ICSI mice (Figure 1, B and C). Litter size and body/placental ratio, which is a measure of placental efficiency, were comparable between the two groups (Figure 1B and Supplemental Table 1). Because ICSI produces offspring with abnormal imprinting and may influence body weight (25), we performed combined bisulfite restriction enzyme analysis (COBRA). We collected tail DNA and determined the DNA methylation levels of DMRs in *H19*, *Meg3* IG, *Igf2r*, and *Snrpn*. None of the mice showed abnormalities (Supplemental Figure 2A). Bisulfite sequencing confirmed these results (Supplemental Figure 3A).

### Behavior analysis of F_1_ animals.

To examine the functional effect on offspring, we conducted a battery of behavioral tests (26). In this experiment, we used only male mice because no obvious sexual differences in behavior were found in a previous study using offspring born after spermatogonial transplantation (27). We compared 3 groups of male mice: F_1_ offspring produced by ICSI using wild-type sperm (ICSI-F_1_) or sperm from GS cells (GS-F_1_) and control offspring sired by natural mating (control-F_1_) (Figure 1A). Although GS-F_1_ mice were heavier, no difference was found in the grip strength and wire hang tests, and they did not exhibit abnormal sensitivity to a thermal stimulus in hot plate test (Supplemental Figure 4, A–D).

Several tests showed reduced locomotor activity of GS-F_1_ mice. GS-F_1_ mice showed reduced distance traveled in the light/dark transition test (Supplemental Figure 4E). Activity level was significantly lower in 24-hour cage monitoring (Supplemental Figure 4F). An open-field test, which is used to assay general locomotor activity levels, anxiety, and exploration activity, showed a tendency toward less activity in GS-F_1_ mice (Supplemental Figure 4G). GS-F_1_ mice showed reduced vertical behavior, spent less time in the center area compared with other types of mice, and had lower stereotypic counts.

**Figure 2:**
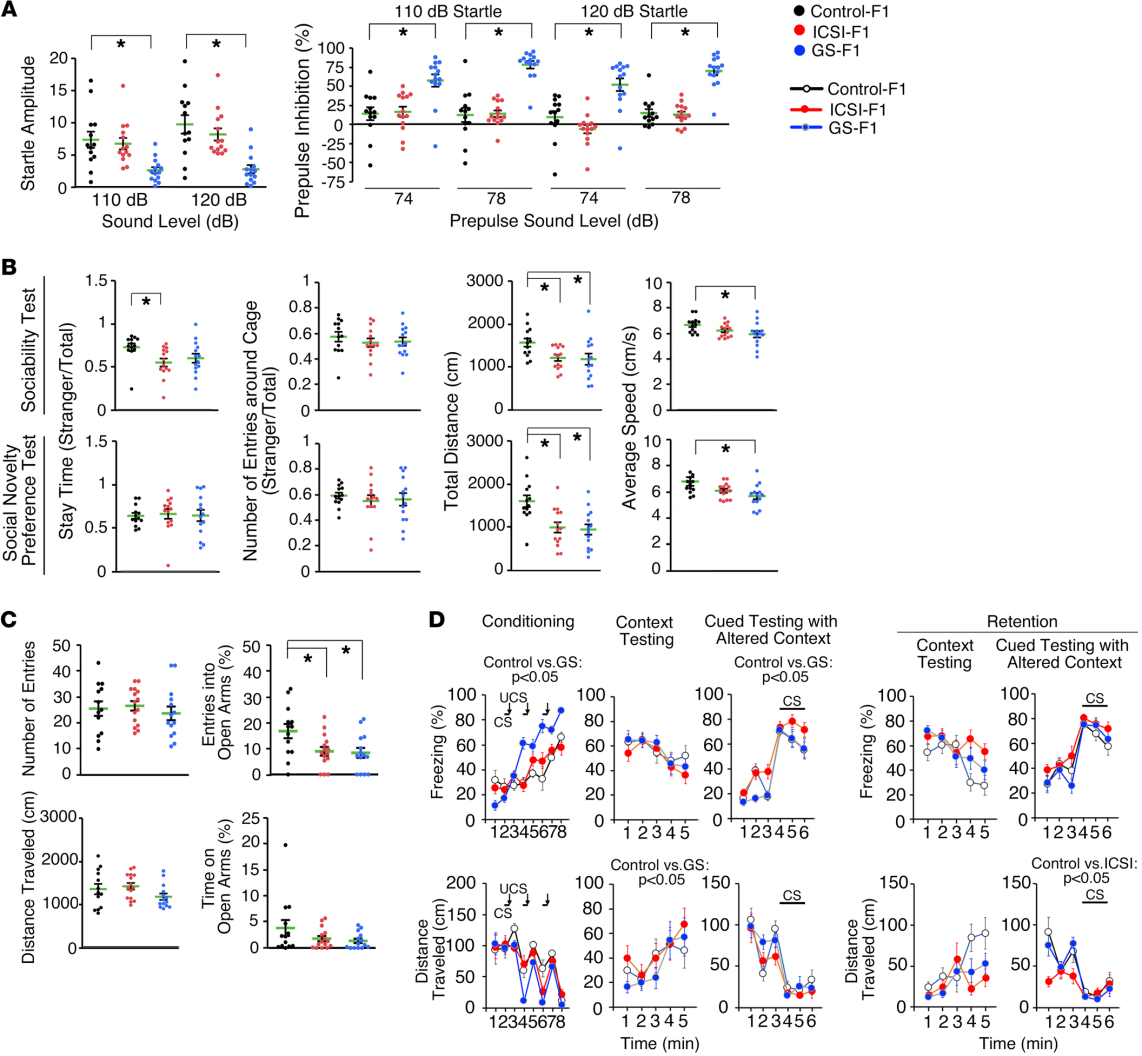
Abnormal behavior of F_1_ offspring. (**A**) Acoustic response and prepulse inhibition test. (**B**) Three-chamber social approach test (Crawley version). In the sociability test, time spent in or around the chamber with an empty cage, the center cage, and the chamber with a stranger mouse (stranger 1) were recorded. In the social novelty preference test, time spent in or around the chamber with a stranger mouse (stranger 1), the center cage, and the chamber with a novel stranger mouse (stranger 2) were recorded. (**C**) Elevated plus maze test. (**D**) Cued and contextual fear conditioning test. The number of mice analyzed is as follows: (**A** and **B**) *n* = 13 for control, *n* = 14 for ICSI-F_1_, and *n* = 14 for GS-F_1_; (**C**) *n* = 13 for control, *n* = 15 for ICSI-F_1_, and *n* = 14 for GS-F_1_; and (**D**) *n* = 13 for control, *n* = 14 for ICSI-F_1_, and *n* = 13 for GS-F_1_. *P < 0.05, 1-way ANOVA (mouse type) or 2-way repeated measures ANOVA (mouse type, 2-way interaction [e.g., mouse type time interaction]). CS, conditioned stimulus; UCS, unconditioned stimulus. See Supplemental Methods and Supplemental Tables 4 and 5 for details.

The most notable characteristic of GS-F_1_ mice was their startle response (Figure 2A). Prepulse inhibition of the acoustic startle response is an index of sensorimotor gating. The startle responses to acoustic stimulation at 110 and 120 dB in GS-F_1_ mice were significantly impaired compared with control mice, suggesting a hearing deficit in GS-F_1_ offspring. However, a weak auditory stimulus at 74 and 78 dB inhibited the startle response more significantly in GS-F_1_ mice, indicating that they do not have a hearing deficit.

Although the tail suspension test showed reduced mobility of GS-F_1_ mice (Supplemental Figure 4H), the Porsolt forced swim test, another test for depressive behavior, showed enhanced immobility and reduced distance traveled (Supplemental Figure 4I). However, because this test also depends on locomotor activity, the result may simply reflect their low locomotive activity. ICSI-F_1_ mice did not show differences in the immobility (i.e., distance traveled); however, several abnormalities were common between ICSI-F_1_ and GS-F_1_ mice. The 3-chamber social approach test (assessing sociability) revealed decreased social behavior in both types of mice (Figure 2B). The sociability test, which compares the behavior around an empty cage and a cage with a stranger mouse (stranger 1), showed that ICSI-F_1_ mice spent less time around the stranger side. Moreover, ICSI-F_1_ and GS-F_1_ mice traveled shorter distance, and the average speed of GS-F_1_ mice was reduced. Although abnormalities in social behavior in GS-F_1_ mice were evident in the social interaction test in a new environment (Supplemental Figure 4J), this test did not show abnormalities in ICSI-F_1_ mice. However, in the elevated plus maze test, which reflects anxiety-like behavior, GS-F_1_ and ICSI-F_1_ mice entered into open arm significantly less frequently (Figure 2C). Therefore, ICSI-F_1_ and GS-F_1_ mice exhibited abnormalities in social behavior and increase in anxiety response.

ICSI-derived F_1_ offspring have impaired memory function (28). To confirm this, we performed several tests. First, the T-maze test, which examines working memory, did not show a defect in ICSI-F_1_ mice (Supplemental Figure 4K). Second, the Barnes maze test, which assesses spatial learning and memory, showed that ICSI-F_1_ mice spent significantly less time around the target hole in probe tests performed 1 month after the last training session and the rate of omission error was significantly increased, suggesting impaired memory retention (Supplemental Figure 4L). A cued and contextual fear conditioning test showed an increase in freezing response and decrease in distance traveled in GS-F_1_ mice in the training session (Figure 2D). GS-F_1_ mice no longer showed abnormalities in the retention test. These results confirmed that ICSI-F_1_ mice have impaired memory.

### Implantation failure and congenital malformation in F_2_ offspring.

To examine whether abnormalities are transmitted to the F_2_ generation, we performed IVF using F_1_ sperm and wild-type oocytes (Supplemental Table 1). After Caesarean section, we found that body and placenta weights of GS-F_2_ mice were significantly increased (Figure 1B). Moreover, the implantation rate was significantly reduced in ICSI-F_2_ mice (Supplemental Table 1). The frequency of placenta-only offspring increased by approximately 16.8-fold when compared with that of ICSI-F_1_ mice. The combined numbers of dead and placenta-only offspring was higher for ICSI-F_2_ offspring, which accounted for approximately 29.6% of newborn offspring. Notably, 8.5% and 1.7% of ICSI-F_2_ offspring exhibited hydrocephalus and anophthalmia, respectively (Figure 1D). Hydrocephalus was also found in 1 GS-F_2_ offspring. Litter size and body/placental ratio were comparable among the 3 groups (Figure 1B and Supplemental Table 1).

We performed another set of IVF using approximately 25-month-old control-F_1_ and ICSI-F_1_ mice to confirm whether ICSI per se causes abnormalities. After Caesarean section, we found that 17.6% of ICSI-F_2_ mice were either dead or placenta only, compared with 3.8% for control-F_2_ mice. Overall, 11.2% of ICSI-F_2_ offspring exhibited congenital malformation. Along with hydrocephalus (1.7%) and anophthalmia (2.6%), offspring were born with small or open eyes (3.4%), skull defect (0.9%), and umbilical hernia (0.9%) were born (Figure 1D).

Unexpectedly, 15.4% of control-F_2_ offspring, which were produced by IVF using sperm from control-F_1_ mice and wild-type oocytes, showed similar congenital deformities (Figure 1D). Offspring with anophthalmia (3.8%), hydrocephalus (3.8%), and small eyes (3.8%) were born. However, the phenotype was not exactly the same because we found 2 offspring with tanned skin (7.7%). Because such abnormalities were not found in control-F_2_ offspring from young control-F_1_ mice (Supplemental Table 1), these results suggested that IVF using aged sperm increases the frequency of congenial malformation.

To determine whether F_1_ female mice can sire abnormal offspring, we performed IVF using ICSI-F_1_ oocytes and wild-type sperm and found an F_2_ offspring with hydrocephalus (Supplemental Table 1). These results showed that congenital abnormalities can occur through the female germline. Based on the increased body weight of GS-F_2_ offspring, we carried out COBRA for all F_2_ offspring (Supplemental Figure 2B); however, no significant abnormalities were found. Bisulfite sequencing confirmed these results (Supplemental Figure 3B).

### Behavioral abnormalities in F_2_ offspring.

To determine whether behavioral abnormalities persist in the F_2_ generation, male F_2_ offspring were subjected to a battery of behavioral tests. Overall, the phenotype of GS-F_2_ mice was stronger than that of ICSI-F_2_ mice. All 3 types of mice had comparable body weights, and no differences were found in a grip strength test and a wire hang test (Supplemental Figure 5, A–C). However, GS-F_2_ mice were more sensitive to heat than control-F_2_ mice (Supplemental Figure 5D).

**Figure 3:**
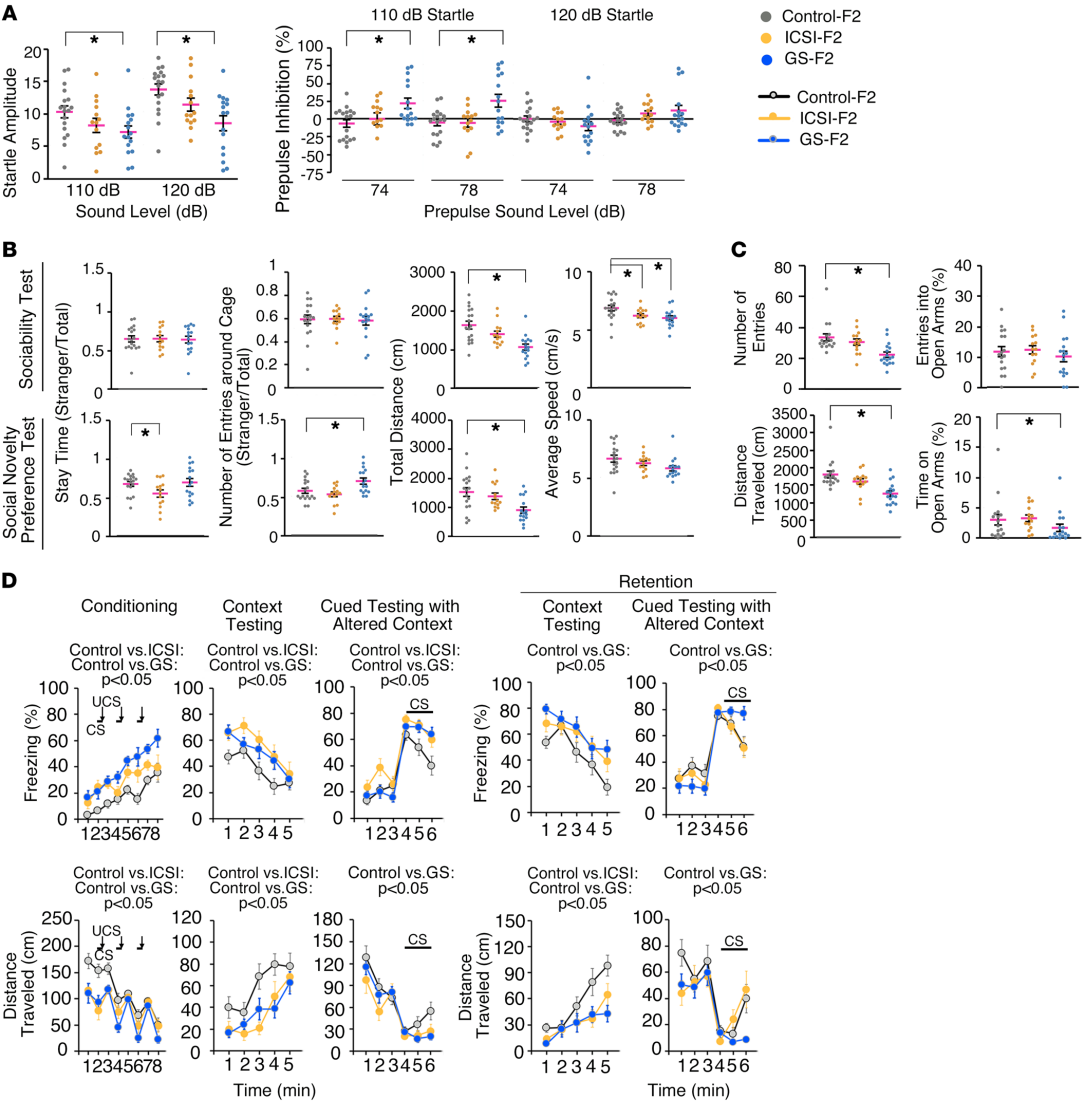
Abnormal behavior of F_2_ offspring. (**A**) Acoustic response and prepulse inhibition test. (**B**) Three-chamber social approach test (Crawley version). (**C**) Elevated plus maze test. (**D**) Cued and contextual fear conditioning test. The number of mice analyzed is as follows: (**A**, **B**, and **D**) *n* = 18 for control-F_2_, *n* = 14 for ICSI-F_2_, and *n* = 16 for GS-F_2_ and (**C**) *n* = 17 for control-F_2_, *n* = 14 for ICSI-F_2_, and *n* = 17 for GS-F_2_. *P < 0.05, 1-way ANOVA (mouse type) or 2-way repeated measures ANOVA (mouse type, 2-way interaction [e.g., mouse type time interaction). CS, conditioned stimulus; UCS, unconditioned stimulus. See Supplemental Methods and Supplemental Tables 4 and 5 for details.

GS-F_2_ mice exhibited many of the defects of GS-F_1_ animals. They showed low activity in the light/dark transition test (Supplemental Figure 5E). Although no abnormalities in 24-hour cage monitoring was found (Supplemental Figure 5F), an open-field test showed lower activity (Supplemental Figure 5G). Abnormalities in acoustic startle response and prepulse inhibition clearly persisted in GS-F_2_ mice (Figure 3A). Despite the lack of a significant differences in the tail suspension test (Supplemental Figure 5H), we found abnormalities in the Porsolt forced swim test (Supplemental Figure 5I). The 3-chamber social approach test and social interaction test in a new environment indicated defective social behavior in GS-F_2_ mice (Figure 3B and Supplemental Figure 5J). Abnormalities in elevated plus maze test also suggested anxiety-like behavior (Figure 3C).

We observed new phenotypes in GS-F_2_ mice. In addition to thermal sensitivity, GS-F_2_ mice showed abnormalities in social novelty preference test (Figure 3B). They also showed a superior response in the T-maze test (Supplemental Figure 5K). Moreover, GS-F_2_ mice showed significant reductions in distance traveled and in number of errors in the Barnes maze test (Supplemental Figure 5L). Therefore, although GS-F_2_ mice exhibited many of the same abnormalities of the GS-F_1_ mice, their memory was significantly improved in the next generation.

The phenotype of ICSI-F_2_ mice was mild. However, they showed abnormalities in the 3-chamber social approach test of social novelty preference (Figure 3B). Control-F_2_ and GS-F_2_ mice spent more time in and around the cage with a new stranger mouse (stranger 2) than in the cage with the familiar mouse (stranger 1), while ICSI-F_1_ mice did not show such a preference. Like GS-F_2_ mice, ICSI-F_2_ mice also exhibited phenotypes not found in ICSI-F_1_ mice. ICSI-F_2_ mice showed a reduction in distance traveled in the dark (Supplemental Figure 5E), showing low locomotive activity. They also showed reduced travel speed in 3-chamber social approach test (Figure 3B). Neither the T-maze test nor Barnes maze test showed abnormalities (Supplemental Figure 5, K and L). However, ICSI-F_2_ mice exhibited a longer freezing time and shorter distance traveled in the conditioning session (Figure 3D). Although the effect of reduced activity needs to be considered, abnormalities were also found in context testing and cued testing with altered context. When fear memory was assessed after 1 month, ICSI-F_2_ mice still showed defects in context testing, suggesting poor learning ability and memory retention (Figure 3D). Therefore, behavioral abnormalities are propagated by germline transmission.

### Congenital deformity in F_3_ offspring.

We produced F_3_ offspring using sperm from F_2_ mice and wild-type oocytes. After Caesarean section, we found that GS-F_3_ offspring were heavier than control-F_3_ mice (Figure 1B). Litter size and body/placental ratio were comparable among the 3 groups (Figure 1B and Supplemental Table 1). Anophthalmia and hydrocephalus were similarly observed in ICSI-F_3_ mice (1.8%; Figure 1E). Moreover, ICSI-F_3_ and GS-F_3_ offspring showed severe defects, with missing head and limbs (Figure 1E). Anophthalmia was found in control-F_3_ offspring (Figure 1E). To examine whether natural mating can erase abnormalities, we crossed ICSI-F_2_ male and female mice with normal appearance. However, natural mating produced 1 mouse with microphthalmia and 1 with hydrocephalus (Figure 1F). COBRA of tail DNA did not show apparent abnormalities in DNA methylation levels (Supplemental Figure 2C). These results suggested that congenital abnormalities occur in the subsequent generations.

### Analysis of spermatogenesis and SSCs in F_1_ mice.

**Figure 4:**
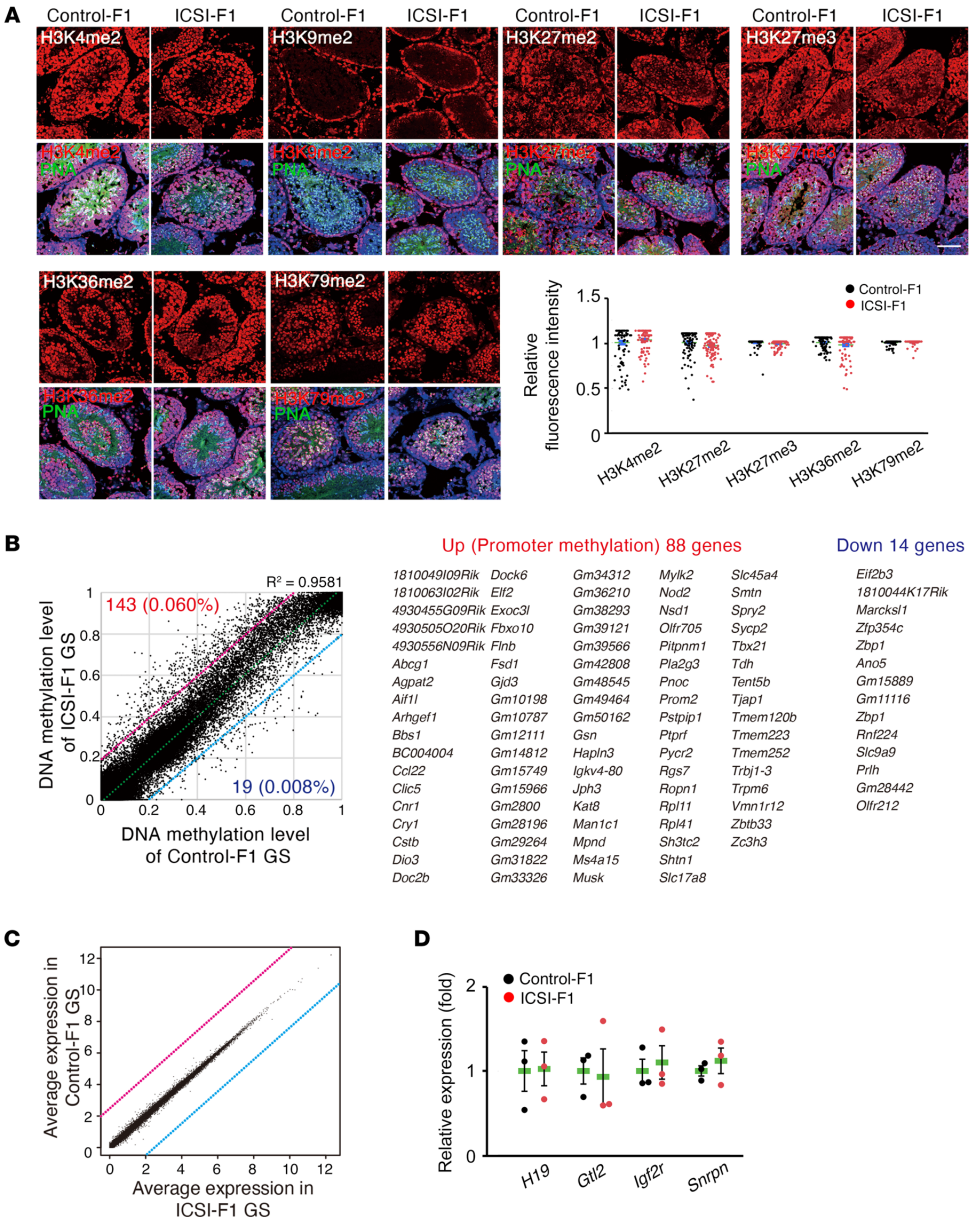
Analysis of spermatogenesis and GS cells derived from F_1_ mice. (**A**) Immunostaining of F_1_ testes using antibodies against H3K4me2, H3K9me2, H3K27me2, H3K27me3, H3K36me2, and H3K79me2. One hundred cells in 5 tubules of 3 mice were analyzed per group. Each antigen was assessed using a single antibody. Signal intensity in PNA^+^ cells was measured. H3K9me2 was omitted for quantification because PNA^+^ cells did not show H3K9me2 signals. Scale bar: 30 μm. (**B**) A scatter plot with a list of genes, showing correlation of the DNA methylation data at individual CpG sites in gene promoters (*n* = 4). Methylation statuses at 237,680 CpG sites were covered. The numbers of identified hypermethylated sites and hypomethylated sites in ICSI-F_1_ compared with control-F_1_ GS cells are shown in red and blue, respectively, along with the percentage of commonly covered sites. Red or blue lines indicate 20% increased methylation levels or 20% decreased methylation levels in ICSI-F_1_ GS cells, respectively. The dashed line indicates the linear regression line. Up, upregulation; Down, downregulation. (**C**) A scatter plot of gene expression by RNA-Seq (*n* = 4). (**D**) Real-time PCR analysis of F_1_ GS cells (*n* = 3). See Supplemental Tables 6 and 7 for details.

To understand the mechanism of transmission of abnormal phenotype, we performed immunostaining of ICSI-F_1_ and control-F_1_ mouse testes (Figure 4A). We used antibodies against the regions of histone H3 containing the dimethylated lysine 4 (H3K4me2), dimethylated lysine 9 (H3K9me2), dimethylated lysine 27 (H3K27me2), trimethylated lysine 27 (H3K27me3), demethylated lysine 36 (H3K36me2), and dimethylated lysine 79 (H3K79me2). Immunostaining pattens were similar to results reported in previous studies (29–31). However, there were no obvious differences in staining patterns between the 2 groups.

To study gene expression in the germline directly, we derived GS cells from ICSI-F_1_ and control-F_1_ mice. GS cells were derived by collecting CD9-expressing spermatogonia from mature testes by magnetic cell sorting. These cells are enriched for SSCs (32). The morphology and growth characteristics of ICSI-F_1_ and control-F_1_ GS cells did not show apparent differences. To study the genomic imprinting in both types of GS cells, we performed COBRA. However, all of them showed the same androgenetic DNA methylation patterns (Supplemental Figure 2D).

We then used the reduced representation bisulfite sequencing method to verify the overall genomic methylation (Figure 4B). Of the 237,680 covered CpG sites, our analysis identified 143 (0.06% of commonly covered sites) hypermethylated sites and 19 (0.008% of commonly covered sites) hypomethylated sites in the ICSI-F_1_ versus the control-F_1_ GS cells (>20% change, R^2^ = 0.9581) (Supplemental Table 2). Gene ontology analysis failed to detect significant association with specific biological functions. Moreover, we were not able to find significant differences in DNA methylation patterns for imprinted genes (Supplemental Figure 6).

We performed RNA-Seq of GS cells for changes in gene expression profiles (Figure 4C and Supplemental Table 3). Comparison between ICSI-F_1_ and control-F_1_ GS cells revealed no differentially expressed genes, including DMR genes (FDR < 0.05). Real-time PCR analysis confirmed comparable levels of imprinted gene expression in both types of F_1_ GS cells (Figure 4D). These results are consistent with the RNA-seq data that showed comparable expression levels of imprinted genes between the 2 cell types.

## Discussion

We found several defects in GS cell–derived F_1_ offspring. Although the animals did not show congenital defects, their bodies were larger, and they exhibited several behavioral abnormalities. In a recent study, several types of behavioral reflexes were analyzed in SSC-derived offspring produced by natural mating using DBA/2 mice (27); however, none of them showed abnormalities in both F_1_ and F_2_ generations. Because SSCs were similarly cultured in that study, abnormalities found in the current study might have been due to difference in genetic background or ICSI. In addition, MHY1485, which was used to drive self-renewal of B6-GS cells in vitro, may also be responsible. Considering that normal offspring were born after spermatogonial transplantation in that study, transplantation procedure per se probably does not play a significant role in inducing abnormalities.

We then found F_2_ offspring produced by IVF using sperm from ICSI-F_1_ mice were abnormal. These results were unexpected because F_1_ offspring appeared normal. However, congenital abnormalities appeared only after germline transmission. Although ICSI-induced transcriptional changes disappear by 8 weeks in somatic cells (33), germ cells of ICSI-F_1_ mice might have undergone irreversible damages, resulting in an increased incidence of abnormalities. For example, whereas the spontaneous rate of hydrocephalus in wild-type B6 mice is 0.029% (ref. 34), the rate of hydrocephalus in F_2_ offspring (~4.0%) was approximately 137.9-fold higher. Although we did not find a statistically significant increase in congenital abnormalities in F_3_ offspring, this was because control-F_3_ offspring, which were produced by 2 rounds of IVF, showed similar defects. Therefore, we currently cannot completely exclude the possibility that congenital abnormalities persist in subsequent generations.

The most likely candidate responsible for inducing abnormal phenotype is acrosome. It has been suggested that incorporation of acrosome into the oocyte by ICSI is hazardous to embryos because acrosome contains an array of hydrolyzing enzymes (35). After fertilization, such enzymes may damage proteins that normally protect DNA. However, because we also observed abnormal offspring after IVF using aged sperm, acrosome alone cannot sufficiently explain the defects. We speculate that atmospheric oxygen may be primarily responsible for the observed phenotype. The concentration of oxygen in vivo varies between 2% and 8% in the oviduct and uterus (36). The atmospheric oxygen is injurious through the generation of free oxygen radicals. Indeed, when pronucleate mouse oocytes were exposed to 20% oxygen for only 1 hour before being cultured in 5% oxygen, there was pronounced inhibition of development (37). Besides oxygen, genetic background may play a role, because our routine ICSI experiments using B6 sperm and B6 × DBA/2 F_1_ (B6D2F1) oocytes do not cause such frequent abnormalities (38).

While these candidate factors need to be tested for potential involvement, our analysis of ICSI offspring and GS cells failed to provide strong evidence for imprinting defects. Because abnormal genomic imprinting can occur after ICSI (25), we focused on imprinted gene expression patterns throughout our analyses. However, none of the imprinted genes showed apparent abnormalities. Moreover, we failed to find significant changes in mRNA expression among F_1_ GS cells. However, more studies are necessary to exclude the possible epigenetic defects; it is possible that in vitro cultures might have influenced epigenetic changes due to exposure to high concentration of oxygen. Considering the many reports on epigenetic defects after animal and human ICSI, we still cannot discount epigenetics as a source of the abnormal phenotype, and analysis of placentas may hopefully provide a clue. Although we failed to show significant differences in the body/placental ratio in newborn offspring, placentas are sensitive to epigenetic abnormalities, and placenta-only offspring are quite often found after nuclear cloning experiments (39). In addition, the possibility of genetic mutations needs to be pursued. It is generally considered that ICSI does not alter mutation frequency (40), and this point is now being analyzed in human samples (41, 42). However, studies using inbred mice may solve this problem more easily. Future studies are required to determine the mechanism of transgenerational defects.

ICSI-induced behavioral abnormalities of F_1_ offspring have been reported in mice (28, 43). Our results were similar, if not identical, to those of these studies. In the current study, we examined the behavior of F_2_ offspring. Although we expected that abnormalities of F_1_ offspring would disappear in F_2_ mice, some abnormalities continued while different phenotypes appeared, despite germline transmission. For example, several abnormalities found in GS-F_1_ mice (anxiety- or depressive-like behavior and social activity), which may reflect their low locomotive activity, continued in the F_2_ generation. However, GS-F_2_ mice showed enhanced spatial learning and working memory, which were not found in GS-F_1_ mice. Moreover, unlike ICSI-F_1_ mice, ICSI-F_2_ mice showed low locomotive activity. Such differences may occur because the F_1_ phenotype reflects ICSI-induced damages in somatic cells. It is not surprising that potentially damaged F_1_ germ cells may produce offspring with distinct properties in the F_2_ generation. It also should be noted that the F_2_ phenotype may reflect the effect of additional IVF.

Our experimental model will be useful to study the science of ART. To date, few models exist to study ICSI-induced damages. We currently do not know whether our results using mice reflect human ARTs because the human acrosome is small and the human oocyte is large compared with those of mice (44, 45). Therefore, the human oocyte may tolerate potential damages caused by acrosomal enzymes. However, the current model using B6 oocytes will provide a useful system to allow improvements of culture conditions and manipulation protocols to minimize ICSI-induced damages. In addition, transgenerational effects of IVF need to be analyzed using larger sample sizes because we found similar defects when we used aged sperm. For application of SSCs, more studies are clearly required. Because offspring derived from GS cell cultures exhibited unique defects (i.e., large body size and startle response), it is likely that GS cell cultures have induced abnormal phenotype. However, it is also possible that offspring production by natural mating may overcome such problems. Because mice have a short generation time with defined genetic backgrounds, such studies will delineate potential hazardous factors and contribute to improve the safety of human ARTs.

## Methods

Further information can be found in Supplemental Methods.

### Animals and transplantation procedure.

We used green mice to derive GS cells (gift from M. Okabe; Osaka University, Osaka, Japan) (46). DBA-GS cells were previously described (22). For analysis of GS cells from F_1_ mice, SSCs were enriched by magnetic cell sorting using anti-CD9 antibody (KMC8; BD Biosciences as previously described (32). For spermatogonial transplantation, B6-GS cells were dissociated with trypsin and microinjected into the seminiferous tubules of 4- to 6-week-old W mice (Japan SLC, Shizuoka, Japan) via the efferent duct (47). Approximately 4 × 10^5^ cells were transplanted into the seminiferous tubules. Each injection filled approximately 75%–85% of the seminiferous tubules.

### IVF and ICSI.

IVF was carried out using human tubal fluid (HTF) medium supplemented with 1.25 mM reduced glutathione, as described previously (48–50). In brief, spermatozoa from epididymis were preincubated in HTF medium at 37°C under 5% CO_2_ in air for 1–2 hours, and a small drop of sperm suspension was added to HTF drops containing cumulus-oocyte complexes. Eggs were collected from C57BL/6N mice, and washed for 4–6 hours after insemination. ICSI was carried out in HEPES (10.1 mM)-CZB medium using a piezo-micropipette-driving unit, as described previously (24, 51). Sperm were collected from testes of recipient W mice and untreated green mice, which were the control. Embryos were cultured for 24 hours in CZB medium at 37°C in an atmosphere of 5% CO_2_ in air and transferred into the oviducts of day 1 pseudopregnant mothers after sterile mating with vasectomized males. All embryo cultures in the present study were performed for 24 hours. Recipients were injected subcutaneously with 2 mg progesterone in the evening on days 18 and 19 to prevent natural delivery. On the morning (09:00–12:00) of day 20, the recipient female mice were examined for the presence of live fetuses by Cesarean section. All analyses were carried out in a nonblinded fashion.

### Statistics.

Significant differences between means for single comparisons were determined by 2-tailed Student’s *t* test. Embryonic development was analyzed using the χ^2^ test. For behavioral tests, either 1-way ANOVA (mouse type) or 2-way repeated measures ANOVA (mouse type, 2-way interaction [e.g., mouse type × time interaction]) was applied. *P* < 0.05 was considered to be significant. Data are shown as the mean ± SEM.

### Study approval.

The Institutional Animal Care and Use Committees of Kyoto University and Fujita Health University approved all animal experimentation protocols.

### Data availability.

Reduced representation bisulfite sequencing and RNA-Seq data have been deposited in the Gene Expression Omnibus (GSE229929 and GSE214649, respectively).

## Author contributions

MKS and TS designed research studies. MKS, YS, NO, KI, SH, KM, NW, AH, KM, TM, and AO conducted experiments. MKS, YS, NO, KI, SH, KM, NW, AH, KM, TY, TM, AO, and TS acquired data. MKS, YS, and TS wrote the manuscript. MKS and YS contributed equally to data acquisition.

## Supplementary Material

Supplemental data

Supplemental tables 1-7

Supporting data values

## Version 1 11/15/2023

Electronic publication
