# Supplementary material for: Intracytoplasmic sperm injection induces transgenerational abnormalities in mice
Source: J Clin Invest. 2023 Nov 15;133(22):e170140. doi: 10.1172/JCI170140 (PMC10645388; doi:10.1172/JCI170140)
Supplement: Supplemental data [file jci-133-170140-s226.pdf]

Supplemental Figure 1

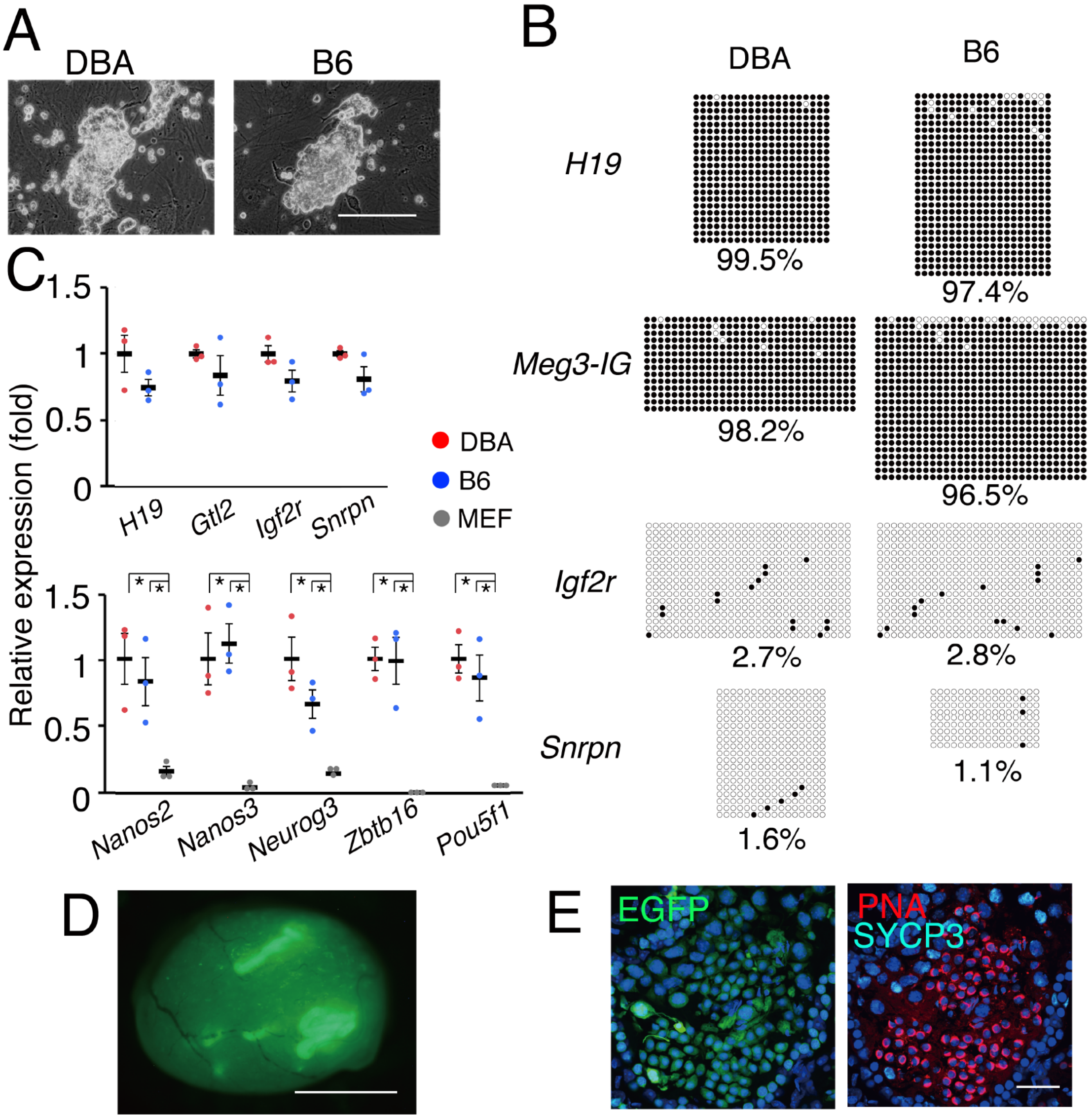

Supplemental Figure 1. Characterization of donor GS cells. (**A**) Appearance of GS cells. (**B**) Bisulfite sequencing of *H19*, *Meg3*-IG, *Igf2r* and *Snrpn* DMRs. (**C**) Real-time PCR analysis of imprinted genes and spermatogonia markers (n = 3). (**D**) Macroscopic appearance of W recipient testis. (**E**) Immunostaining of recipient testis by meiotic (SYCP3) and haploid cell (PNA) markers. Bar = 50 μm (A), 2 mm (D), 30 μm (E). Asterisk indicates statistical difference by 2-tailed Student's t-test ($p < 0.05$).

Supplemental Figure 2

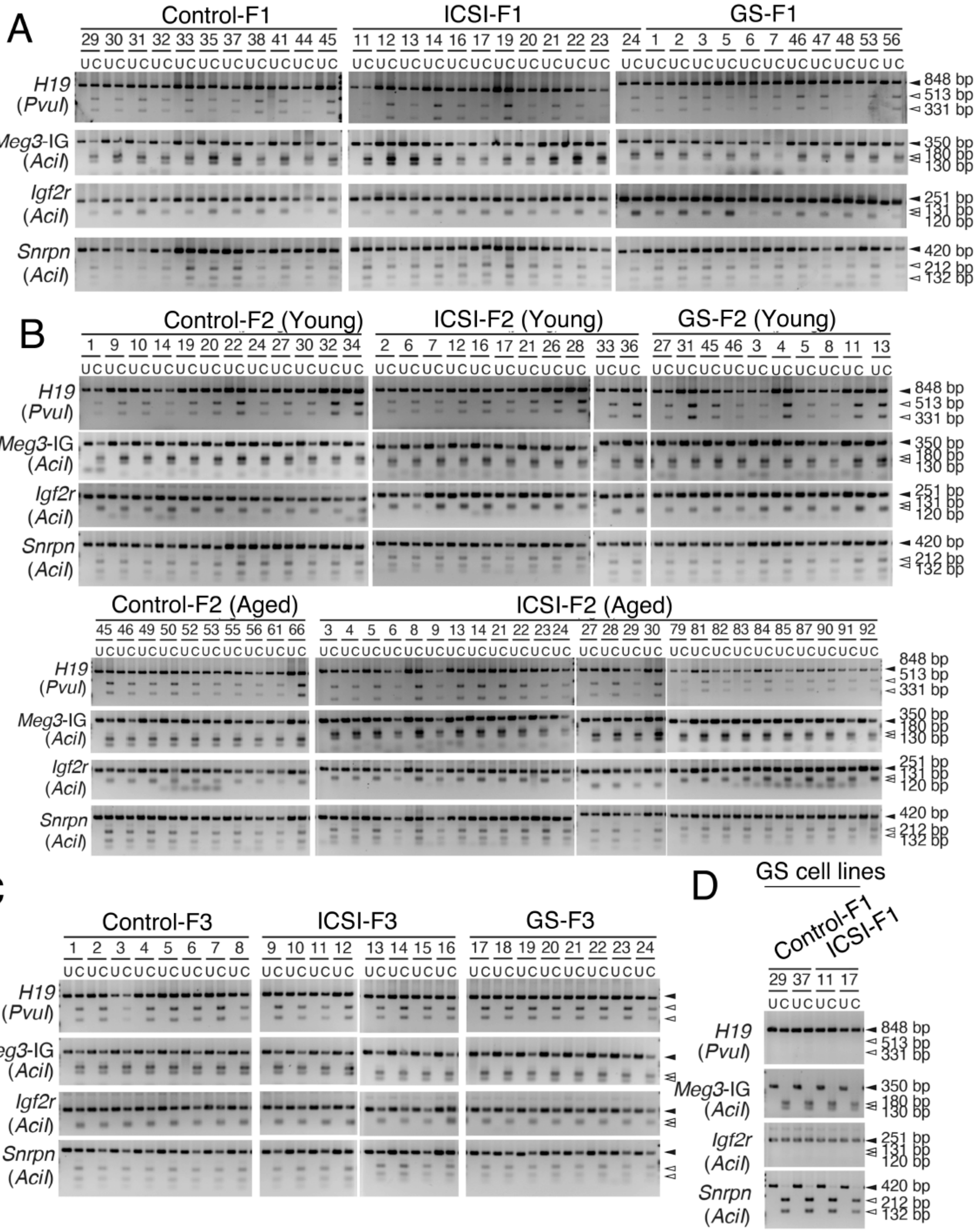

Supplemental Figure 2. COBRA of tail DNA. (**A**) F1 offspring. (**B**) F2 offspring. (**C**) F3 offspring. (**D**) GS cells from F1 mice.

Supplemental Figure 3

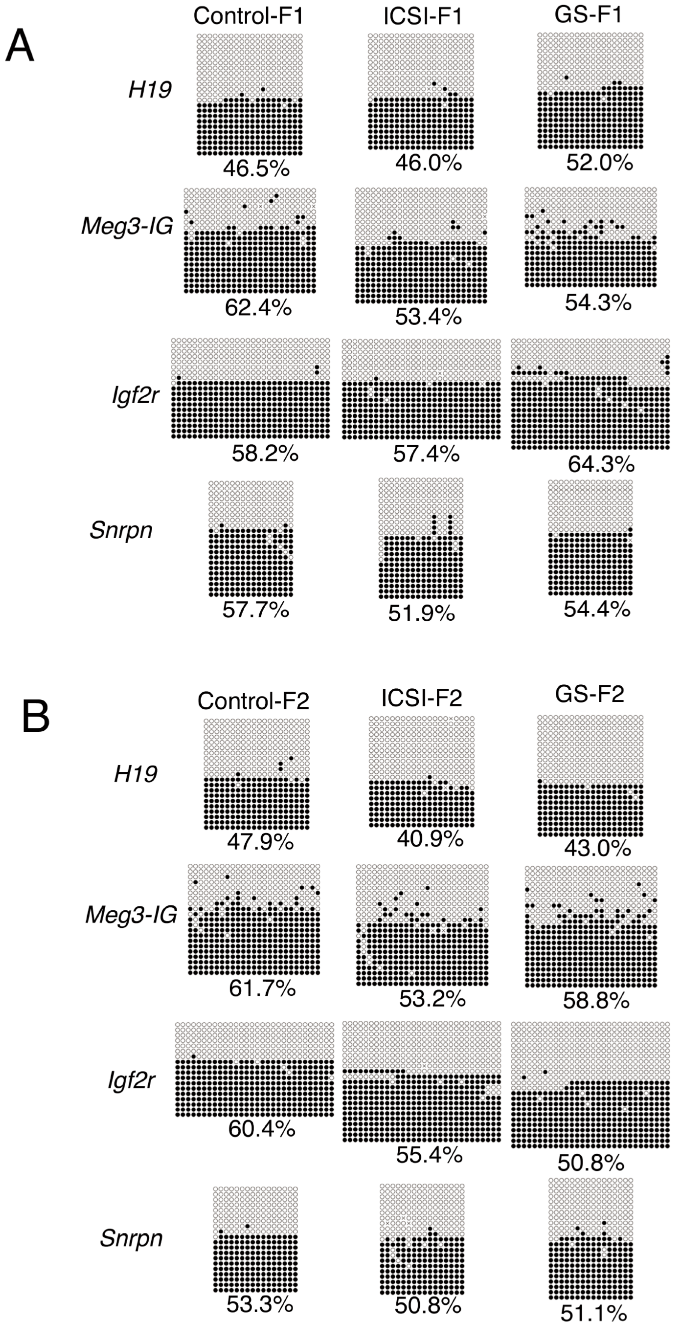

Supplemental Figure 3 Bisulfite sequencing of tail DNA. (**A**) F1 offspring. (**B**) F2 offspring.

Supplemental Figure 4

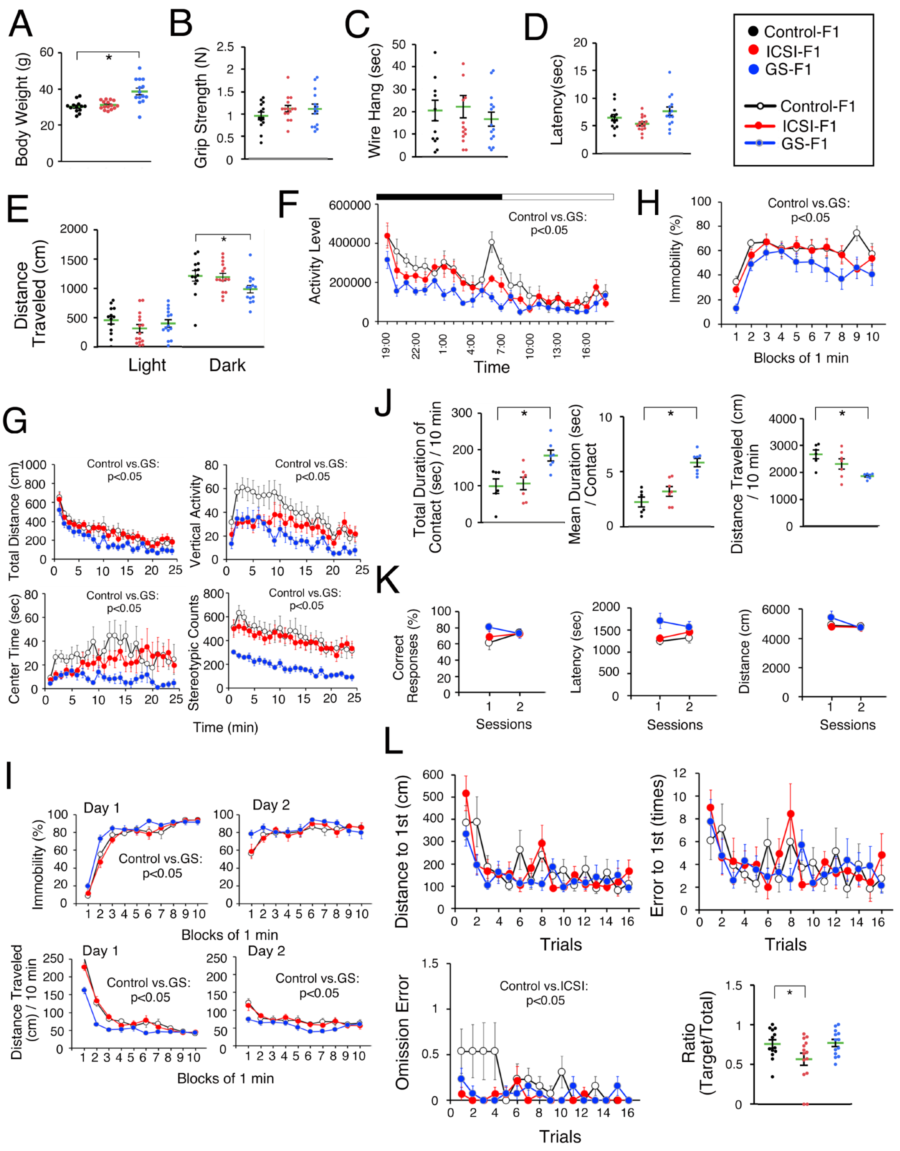

Supplemental Figure 4. Behavior test battery of F1 mice. (A) Body weight. (**B**) Grip strength test. (**C**) Wire hang test. (**D**) Hot plate test. **(E)** Light/dark transition test. **(F)** 24 h home cage monitoring. **(G)** Open field test. (**H**) Tail suspension test. **(I)** Porsolt forced swim test. (**J**) Social interaction test in a new environment. (**K**) T-maze test. (**L**) Barnes maze test. The number of mice analyzed: (A-E, G) n=13 for control; n=15 for ICSI-F1; n=14 for GS-F1. (F) n=5 for control; n=6 for ICSI-F1; n=6 for GS-F1. (H, L) n=13 for control; n=14 for ICSI-F1; n=13 for GS-F1. (I) n=13 for control; n=14 for ICSI-F1; n=14 for GS-F1. (J) n=6 for control; n=7 for ICSI-F1; n=7 for GS-F1. (K) n=13 for control; n=14 for ICSI-F1; n=14 for GS-F1. Asterisk indicates statistical difference by one-way ANOVA (mouse type) or two-way repeated measures ANOVA [mouse type, 2-way interaction (e. g. mouse type × time interaction)]($p < 0.05$).

Supplemental Figure 5

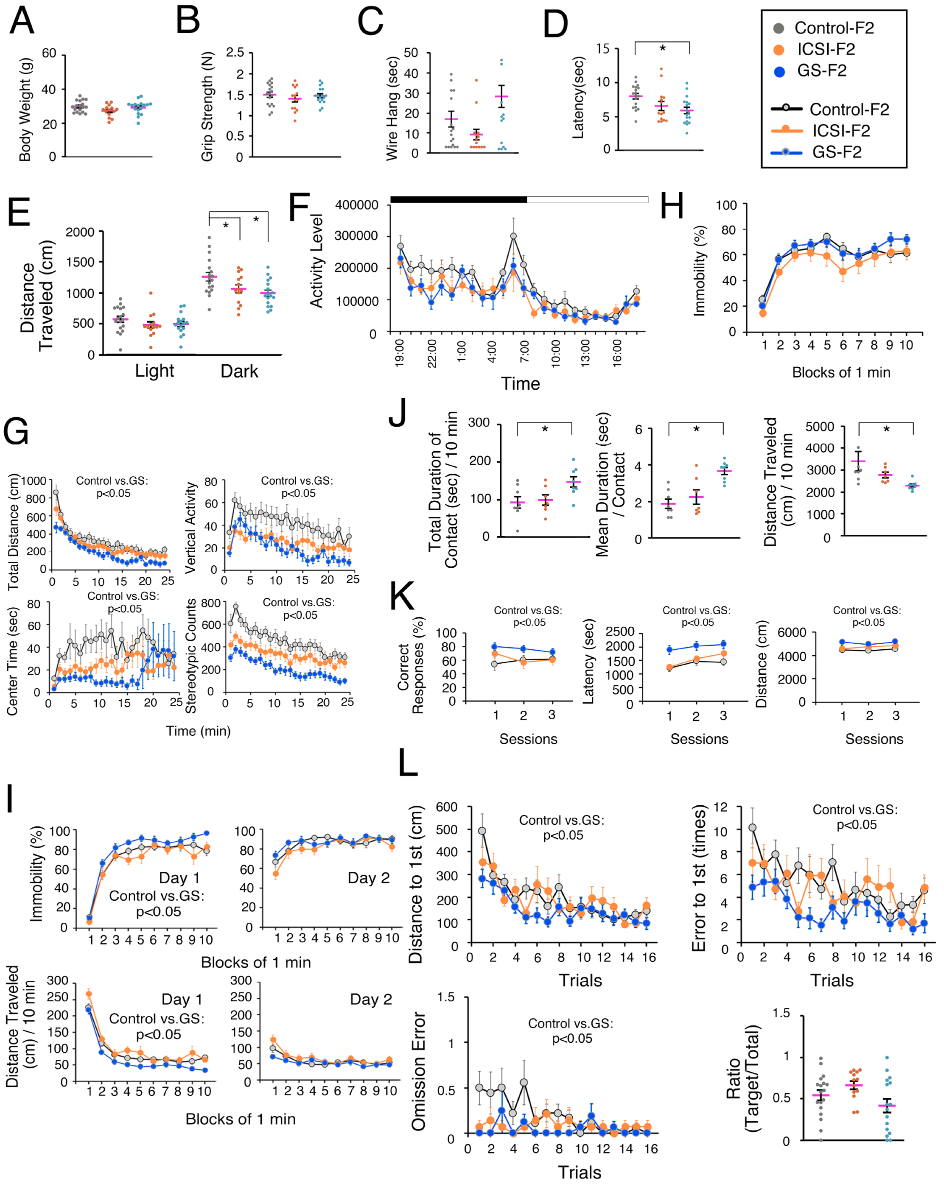

Supplemental Figure 5. Behavior test battery of F2 mice. (A) Body weight. (**B**) Grip strength test. (**C**) Wire hang test. (**D**) Hot plate test. **(E)** Light/dark transition test. (**F**) 24 h home cage monitoring. **(G)** Open field test. (**H**) Tail suspension test. **(I)** Porsolt forced swim test. (**J**) Social interaction test in a new environment. (**K**) T-maze test. (**L**) Barnes maze test. The number of mice analyzed: (A-E, G), n=18 for control-F2; n=14 for ICSI-F2; n=17 for GS-F2. (F, J) n=8 for control-F2; n=7 for ICSI-F2; n=8 for GS-F2. (H) n=17 for control-F2; n=13 for ICSI-F2; n=16 for GS-F2. (I) n=17 for control-F2; n=14 for ICSI-F2; n=16 for GS-F2. (K, L) n=18 for control-F2; n=13 for ICSI-F2 of ratio (target/total) and n=14 for others; n=16 for GS-F2. Asterisk indicates statistical difference by one-way ANOVA (mouse type) or two-way repeated measures ANOVA [mouse type, 2-way interaction (e. g. mouse type × time interaction)]($p < 0.05$).

Supplemental Figure 6

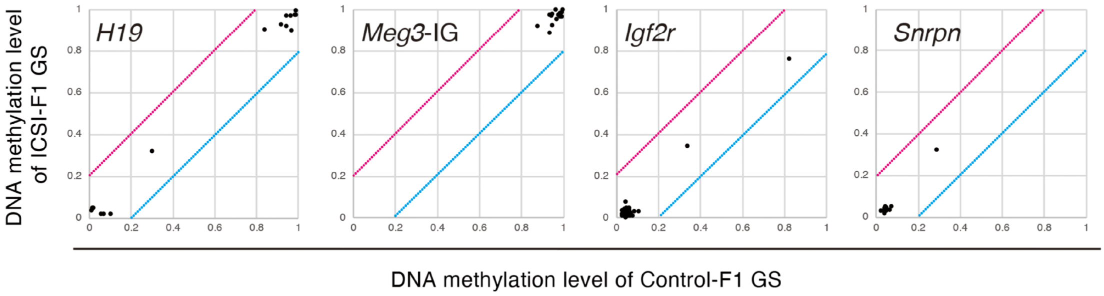

Supplemental Figure 6. DNA methylation patterns in DMRs of imprinted genes. Examples of sequence alignments for DMRs of *H19*, *Meg3*-IG, *Igf2r*, and *Snrpn* genes. Red or blue lines indicate 20% increased methylation levels (hypermethylation) or 20% decreased methylation levels (hypomethylation) in ICSI-F1 GS cells. Each dot indicates individual CpG.

Supplemental Figure 7

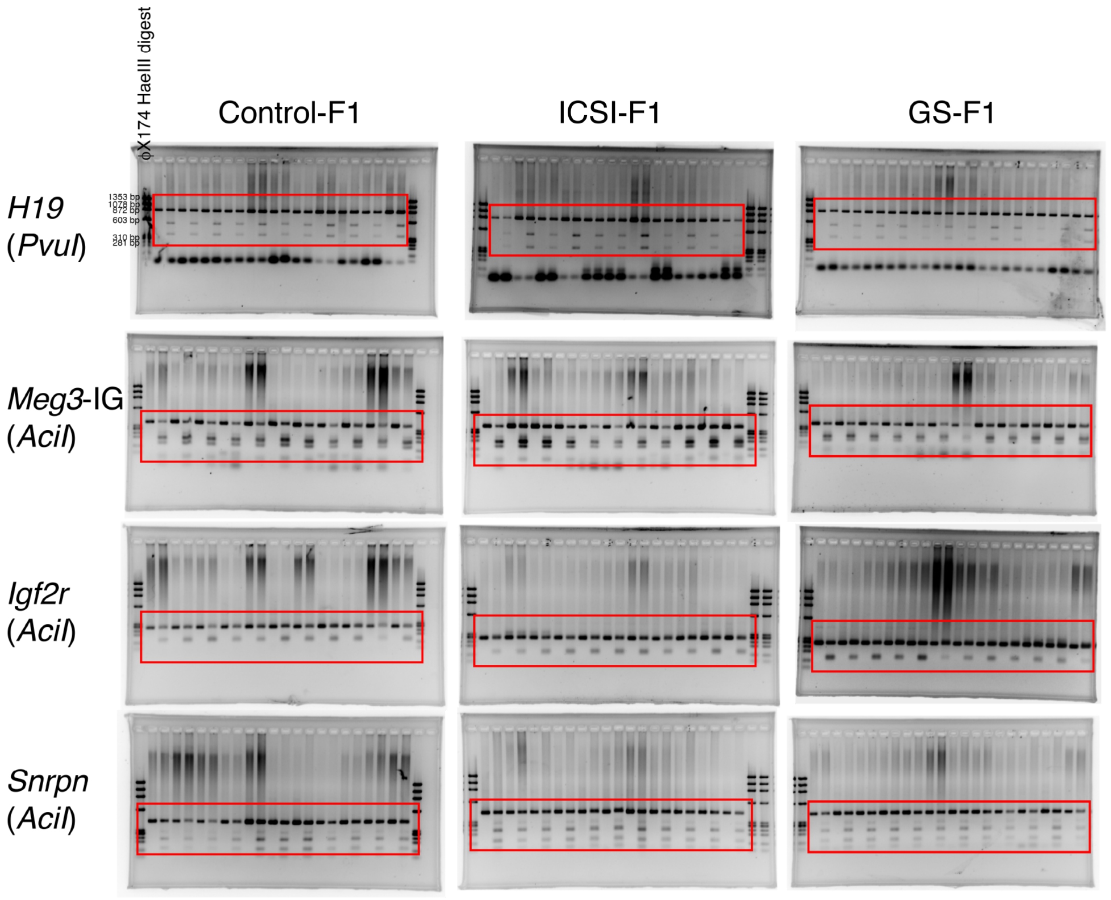

Uncropped gels for Supplemental Figure 2A

Supplemental Figure 7. Uncropped gel images for Supplemental Figure 2A. Red boxes delineate the cropped area of the gel shown in the indicated figure.

Supplemental Figure 8

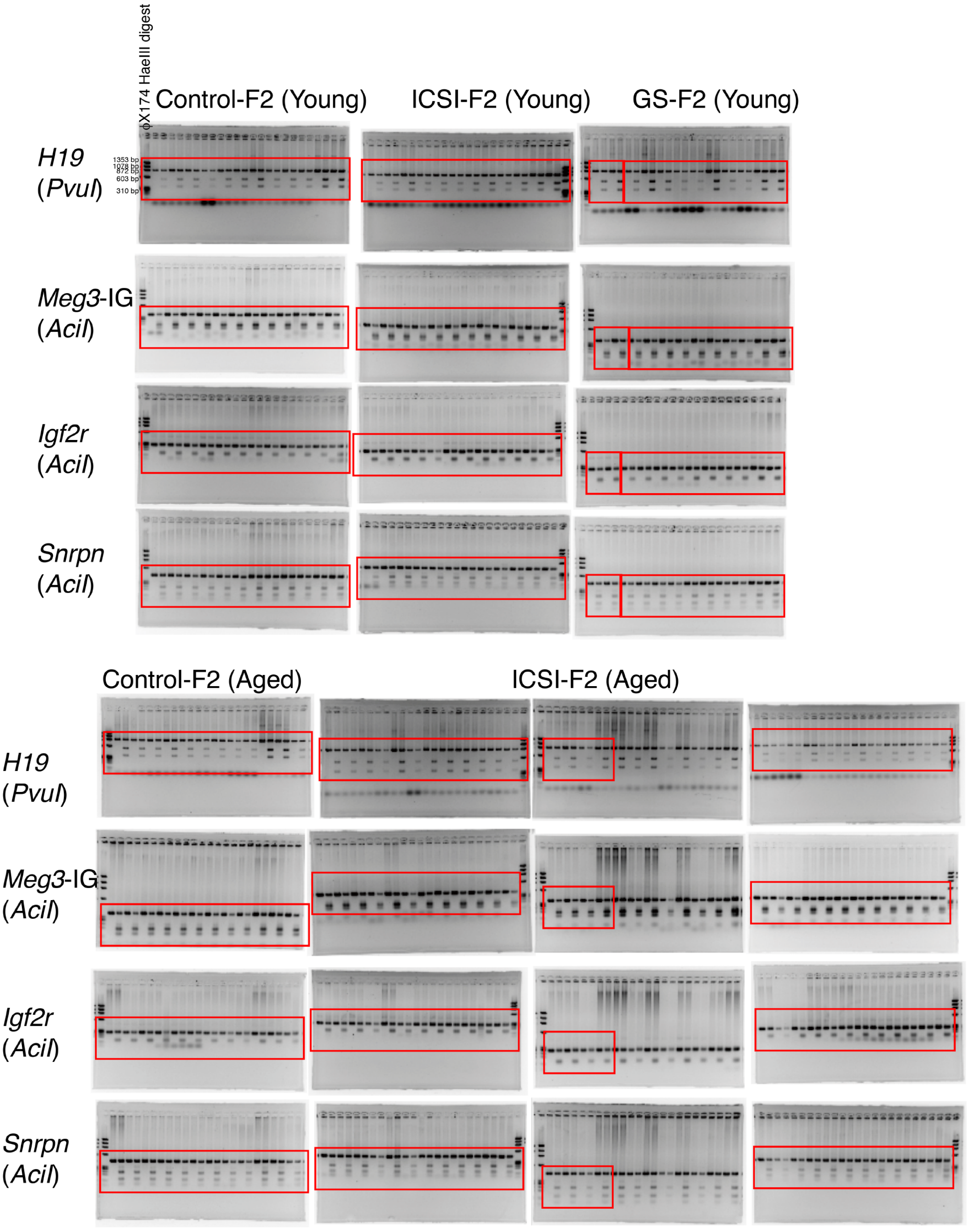

Uncropped gels for Supplemental Figure 2B

Supplemental Figure 8. Uncropped gel images for Supplemental Figure 2B. Red boxes delineate the cropped area of the gel shown in the indicated figure.

Supplemental Figure 9

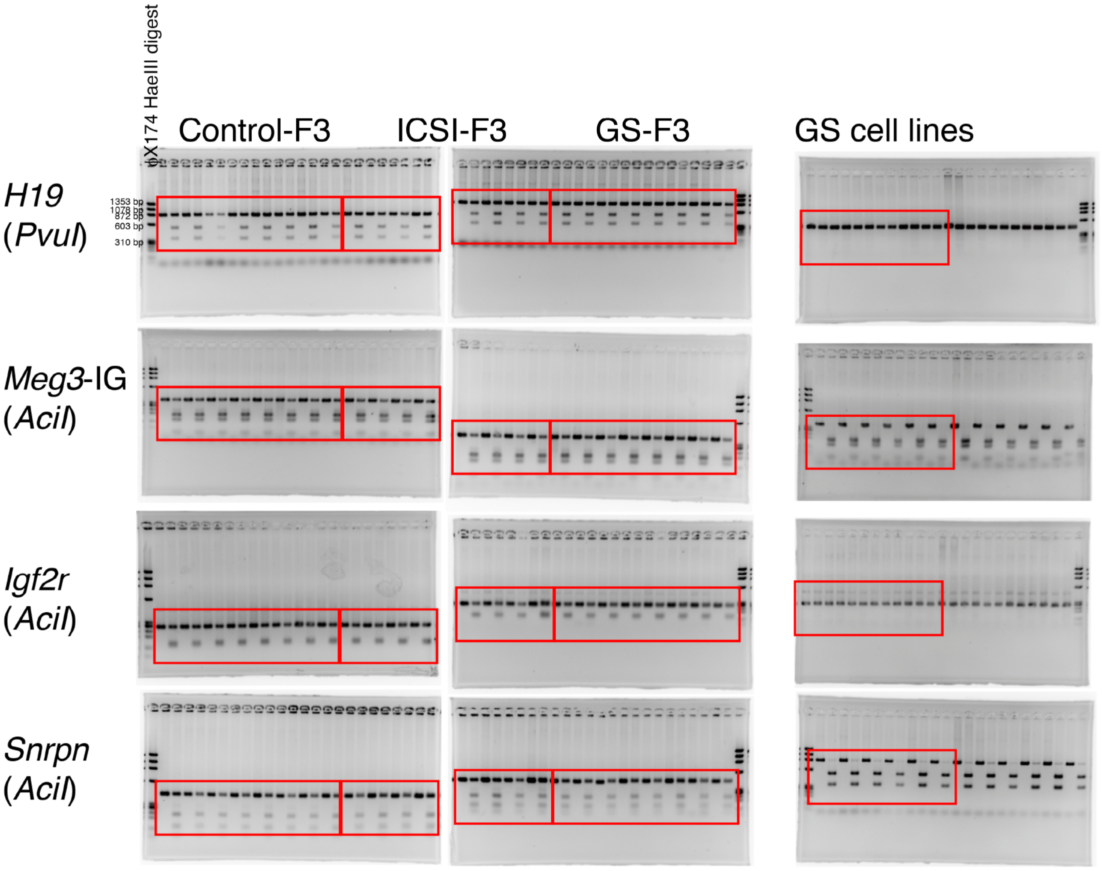

Uncropped gels for Supplemental Figure 2C and 2D

Supplemental Figure 9. Uncropped gel images for Supplemental Figure 2C and D. Red boxes delineate the cropped area of the gel shown in the indicated figures.

Supplemental methods

Experimental design for behavioral experiments

Behavioral analysis was carried out at the Institute for Comprehensive Medical Science, Fujita Health University (Join Usage/Research Center for Genes, Brain and Behavior accredited by Ministry of Education, Culture, Sports, Science and Technology, Japan), as previously described (1). For each type of mouse, we conducted the experiments in two batches (F1 and F2). The number of animals analyzed were 13 to 15 for F1 generation, whereas 14 to 18 animals were used for F2 generation. Composition of the offspring in each group is indicated in Supplemental Table 4. The mice in each group were regrouped at least one month prior to the start of testing, and group-housed in a room with a 12-h light, 12-h dark cycle (lights on at 7:00) with ad libitum access to food and water. Behavioral tests were performed between 09:00 and 18:00 using protocols identical to previous studies (1). The battery of tests started from mild to more severe studies, and was conducted in the following order: general health and neurological screening, light/dark transition test, open field test, elevated plus maze test, hot plate test, social interaction test in a novel environment, 3-chamber social approach test, startle response/PPI test, Porsolt forced swim test, T-maze test, Barnes maze test, tail suspension test, fear conditioning test, and social interaction test in home cage. To minimize carryover effects from previous test, each test was spaced at least one day apart. The order of trials for each test was counterbalanced between experimental groups. The age of the mice at the time of the experiment are shown in Supplemental Table 5.

Behavioral tests

General health and neurological screening

At the beginning of behavioral test battery, physical characteristics and sensorimotor reflexes were evaluated to ensure the health status and neurological function. The neuromuscular function was assessed by grip strength and wire hang tests. A grip strength meter (O'Hara & Co., Tokyo, Japan) was used to measure forelimb grip strength. Each mouse was positioned to grasp a wire by the forelimbs and gently pulled backward by the tail. The measurements were performed three times, the greatest value obtained was analyzed. In the wire hang test, each mouse was placed on a wire mesh on the top of the apparatus (O'Hara & Co.), and the wire mesh was then slowly inverted. The Latency to fall

was recorded with a 60 sec cutoff time.

Light/dark transition test

The apparatus consisted of two equal-sized plastic chambers (20 × 20 cm; O'Hara & Co.), separated by a partition plate with a small opening. One chamber was brightly illuminated (390 ± 20 lx) and the other was dark (< 2 lx). Each mouse was placed in the dark chamber and allowed to freely explore the apparatus for 10 min. Data recording and analysis were performed automatically using Image LD software.

Open field test

Locomotor activity was measured using an open field apparatus (40 × 40 × 30 cm; Accuscan Instruments, Columbus, OH). The center of the field was illuminated at 100 ± 5 lx. A mouse was placed in the corner of the apparatus and the data were recorded by the VersaMax system for 120 min.

Elevated plus maze test

The apparatus consisted of two open arms (25 × 5 cm; O'Hara & Co.) crossing two enclosed arms of identical size with 15-cm high transparent walls. The maze was elevated 50 cm above the floor and illuminated at 100 lx at the central square (5 × 5 cm). Each mouse was placed at the center of the maze facing one of the closed arms. The behavior was recorded for 10 min, and the analysis were performed automatically using Image EP software.

Hot plate test

Each mouse was placed on a 55°C hot plate (Columbus Instruments, Columbus, OH). The latency to escape from thermal stimulation was recorded with a 15-s cut-off time.

Social interaction test in a novel environment

Two mice of the same experimental group that had been reared in different cages were placed together in a field (40 × 40 × 30 cm; O'Hara & Co.) and allowed to explore freely for 10 min. Data recording and analysis were performed automatically using Image SI software.

3-chamber social approach test

The experimental apparatus consisted of a rectangular box separated into three equal-sized chambers (20 × 40 cm; O’Hara & Co.) by transparent plates with small openings (5 × 3 cm). One wire cylinder-shaped cage was placed at each corner of left and right chambers. The subject mice were first allowed to freely explore the chambers for 10 min as a habituation period. In sociability test, a stranger mouse (stranger 1) was placed in the wire cage on either side, and the position was systematically alternated between trials. In social preference test, a second stranger mouse (stranger 2) was placed in the wire cage in the opposite side chamber. For both tests, the subject mouse was allowed to freely explore the chambers for 10 min. The number of contacts and the amount of time spent with strangers 1 and 2 were automatically recorded and analyzed using Image CSI.

Startle response/PPI test

Each mouse was placed in a cylinder chamber mounted on a movement sensor in a sound-attenuated box (O’Hara & Co.) and left undisturbed for 10 min. A test session was composed of 6 different trial types: two startle stimulus-only trials (110 or 120 dB) and four types of PPI trials (74-110, 78-110, 74-120 or 78-120 dB). Six blocks of the six trial types were presented in a pseudorandom order within each block. The peak startle amplitude was used for analysis. The PPI (%) was calculated for each trial type according to the following equation: [1– (startle amplitude in a PPI trial)]/(startle amplitude in a startle stimulus-only trial)] × 100.

Porsolt forced swim test

The apparatus consisted of a Plexiglas cylinder (22 cm in height and 12 cm diameter; O’Hara & Co.) filled with water (room temperature) up to a height of 7.5 cm. Each mouse was placed in the cylinder, and images were captured for 10 min and automatically analyzed using Image TS software.

T-maze test

The modified T-maze apparatus was constructed of white plastic runways with walls 25-cm high partitioned into 6 areas, the stem of the “T” (starting compartment), a

straight runway, the left and right arms, and passageways from each arm to the stem of the "T" (O'Hara & Co.). One session consisted of 10 trials with a 50-min cutoff time and each trial had one forced run followed by one free run. On the forced run, the mouse was forced to proceed in one direction. After returning to the starting compartment, the mouse was allowed to choose to enter the left or right arm. If the mouse chose the opposite direction from the previous forced run, it was counted as a correct response. Data acquisition and analysis were performed automatically by Image TM.

Barnes maze test

The Barnes maze test was conducted on a white circular open filed (1 m in diameter) with 12 holes equally spaced around the margin, and elevated 81.5 cm from the floor (O'Hara & Co.). A black plastic box (17 × 13 × 7 cm) was positioned under one of the holes (the target hole). The center of apparatus was illuminated at 800 lx or more. In a training session, the mice were placed in the center of the field and freely explore the field with a 5-min cutoff time. Each trial was completed when the mice entered the black plastic box under the target hole. A training trial was conducted over 2 weeks until 16 trials. Probe tests were conducted for 3 min without the black plastic box one day or one month after the final training trial. Data recording and analysis were performed automatically by Image BM software.

Tail suspension test

Mice were suspended 30 cm above the floor by adhesive tape placed approximately 1 cm from the base of the tail. The behavior during suspension was recorded for 10 min using Image TS software.

Fear conditioning test

In the conditioning session, mice were placed in an acrylic chamber (26 × 34 × 33 cm, O'Hara & Co.). The conditioned acoustic stimulus (CS, 55 dB) was presented for 30 s at three times (2, 4, and 6 min after the start), and a mild electric foot shock (2 s, 0.3 mA) was served at the end of each CS as the unconditional stimulus (UCS). One day or one month after the conditioning session, contextual fear was measured in the same chamber for 5 min. After the context test, cued fear was measured for 6 min in a white

opaque triangular box (33 × 33 × 33 cm). Mice were placed in the chamber for 3 min with neither CS nor UCS presented, and the CS (55 dB) was presented for the last 3 min. Data acquisition, control of stimuli (i.e., tones and shocks), and data analysis were conducted automatically using Image FZ software.

Social interaction test in home cage

The 24 h monitoring system for analyzing locomotor activity and social interaction under familiar conditions consisted of a standard home cage (29 × 19 × 13 cm), a filtered cage top and an infrared video camera (O'Hara & Co.). Two mice of the same experimental group that had been housed separately were placed together in the home cage for one continuous week. Data acquisition and analysis were conducted using Image HA software.

Image analysis of behavioral test

The applications used for the behavioral tests (Image LD, Image EP, Image SI, Image CSI, Image TS, Image TM, Image BM, Image FZ, and Image HA) were based on ImageJ (https://imagej.nih.gov/ij/) and modified for each test by Tsuyoshi Miyakawa (available through O'Hara & Co.).

Immunostaining

Testis or placenta samples were fixed in 4% paraformaldehyde for 2 h at 4°C, embedded in Tissue-Tek OCT compound (Sakura Finetek, Tokyo, Japan) for cryosectioning. Sections of 8 μm thickness were made. To block non-specific antibodies, sections were treated with 3% bovine serum albumin and 10% goat or donkey serum in phosphate-buffered saline supplemented with 0.1% Tween 20 (PBST) for 1 h at room temperature. The sections were then incubated with indicated antibodies (Supplemental Table 6). Rhodamine-labeled PNA (RL-1072; Vector Laboratories, Burlingame, CA) was used to detect the acrosome. Hoechst 33342 (Sigma, St. Louis, MO) was used for counterstaining.

To quantify histone modification, immunofluorescent images were acquired by Olympus FV3000 confocal laser microscope (Olympus, Tokyo, Japan). The fluorescence intensity in individual PNA-positive cells was quantified as the average pixel value within a nucleus using the Fiji software (2). The mean of control-F1 was set as 1, and the intensity of the ICSI-F1 was calculated as a relative value.

COBRA

Genomic DNA was treated with sodium bisulfite, which deaminates unmethylated cytosines to uracils but does not affect 5-methylated cytosines. The DNA was used as a template to amplify differentially methylated regions using specific primers (Supplemental Table 7). The PCR product was digested with the indicated restriction enzymes, which recognize DNA sequences containing CpG in the original unconverted DNA. See complete unedited gels in Supplemental Figure 7-9.

Bisulfite sequencing

DNA methylation analysis was performed as described previously using specific primers (Supplemental Table 7) (3). Bisulfite-treated DNA was amplified by PCR using specific primer sets, and the amplified PCR products were purified using FastGene Gel/PCR Extraction kit (NIPPON Genetics, Tokyo, Japan) according to the manufacturer's protocols. DNA methylation analyses were performed using the Quantification Tool for Methylation Analysis (QUMA) (http://quma.cdb.riken.jp/top/quma_main_j.html)).

RRBS

Genomic DNA was extracted from GS cells using the DNeasy Blood and Tissue Kit (Quiagen, Valencia, CA). Genomic DNA libraries were generated using a Zymo-Seq RRBS Library Kit (Zymo Research; Orange, CA). 300ng of genomic DNA were digested with MspI and ligated to RRBS-adaptors. Bisulfite treatment of the fragments was done using the EZ DNA Methylation–Lightning kit. The bisulfite converted DNA fragments were amplified by PCR using index primers. The amplification condition was 94°C for 30 s; 94°C for 30 s, 55°C 30 s, 68°C 60 s, apply for 12 cycles; 68°C 5 min. Sequencing was performed using NovaSeq6000 (Illumina, San Diego, CA) with a paired-read sequencing length of 150 bp. The numbers of reads of Control_F1_C_29, Control_F1_C_37, Control_F1_C_44, Control_F1_C_45, ICSI_F1_M1_11, ICSI_F1_M1_17, ICSI_F1_M1_20, and ICSI_F1_M1_21 were 68,087,100, 63,728,322, 81,362,378, 92,937,406, 65,444,610, 66,436,082, 81,894,858, and 92,414,788, respectively.

To calculate DNA methylation levels in GS cells, sequenced reads were analyzed with the following software. The quality of the raw sequence reads was assessed with FastQC

(Version 0.11.7; https://www.bioinformatics.babraham.ac.uk/projects/fastqc/). Low quality (< 20) bases and adapter sequences were trimmed by TrimGalore (Version 0.5.0) with "-q 20 --phred33 --rrbs --paired --non_directional" options. The trimmed reads were aligned to the mm10 mouse reference genome using Bismark (Version 0.20.0) with the "--non_directional" option. The Bismark-resultant .sam files were converted into .bam files with Samtools (Version 1.9).

Methylated positions were called using the processBismarkAln of methylKit (Version 1.10.0). The number of methylated Cs in 1000 bases of non-overlapping windows across the whole genome was estimated by the tileMethylCounts function from the methylKit package. Normalization was performed using the normalizeCoverage function in methylKit. Bases having below 10 read coverage and bases with more than 99.9th percentile of coverage in each sample (likely PCR artifacts with abnormally high coverage) were removed by the filterByCoverage function of methylKit. All the samples were then merged by using the unite function in methylKit with the destrand = FALSE option. Cytosines/Regions with a methylation change larger than 20% between the groups were considered as significantly differentially methylated cytosines/regions. However, those with reversed DNA methylation changes in any one of the samples were excluded.

The genomic coordinates of CpG islands were downloaded from UCSC (mm10, table:cpgIslandExt). Promoters were defined as genomic regions encompassing 1 kb upstream of the transcriptional start site. The genomic coordinates of transcriptional start sites were downloaded from Ensembl (Mus_musculus.GRCm38.87.processed.gtf). GO analysis was performed using ShinyGO 0.77 tool (http://bioinformatics.sdstate.edu/go/).

Gene expression analysis

Total RNA was isolated using TRIzol reagent (Invitrogen, Carlsbad, CA). For GS cell cultures, cells were incubated on gelatin-coated plates for 2 h to remove embryonic fibroblasts before sample collection. First-strand cDNA was produced using a Verso cDNA Synthesis Kit for reverse transcription-PCR (RT-PCR) (Thermo Fisher Scientific, Waltham, MA). For real-time PCR, the Fast SYBR Green Master Mix (Thermo Fisher Scientific) was used according to the manufacturers' protocols. Transcript levels were normalized to those of *Hprt*. PCR conditions were as follows: 95°C for 10 min, followed by 40 cycles at 95°C

for 15 s and 60°C for 1 min.

For RNA sequencing, total RNA was isolated using an RNeasy Mini Kit (Qiagen,Valencia, CA). RNA integrity was assessed with Bioanalyzer or Tapestation (Agilent Technologies, Santa Clara, CA). The sequencing libraries were generated from 100 ng total RNA using a TruSeq stranded mRNA sample prep kit (Illumina) according to the manufacturer's instructions. Fifteen cycles of PCR were performed for library preparation. Sequencing was performed on an Illumina NextSeq550/500 with a single read sequencing length of 76 bp. The fastq files were generated using bcl2fastq-2.20. The number of reads of Control_F1_C29, Control_F1_C37, Control_F1_C44, Control_F1_C45, ICSI_F1_M1_11, ICSI_F1_M1_17, ICSI_F1_M1_20, and ICSI_F1_M1_21 were 32,252,603, 33,400,287, 43,796,577, 56,729,221, 34,121,642, 34,908,307, 41,894,594, and 44,242,607, respectively. Adapter sequences and low-quality bases were trimmed from the raw reads using cutadapt v3.4 (4). The trimmed reads were mapped to the mm10 mouse reference using STAR ver 2.7.9a with the GENCODE M25 gtf file (5-7). The raw counts were calculated using htseq-count v. 0.13.5 with the GENCODE gtf file. DEGs were identified with fold changes and FDR determined with DESeq2 v1.30.1 (8). Gene expression levels were calculated as Transcripts Per Kilobase Million (TPM) values.

Supplemental references

1. Shoji H, Miyakawa T. Age-related behavioral changes from young to old age in male mice of a C57BL/6J strain maintained under a genetic stability program. *Neuropsychopharmacol Rep.* 2019;39(2):100-118.
2. Schindelin J, et al. Fiji: an open-source platform for biological-image analysis. *Nat Methods.* 2012;9(7):676-682.
3. Kanatsu-Shinohara M, et al. Adeno-associated-virus-mediated gene delivery to ovaries restores fertility in congenital infertile mice. *Cell Rep Med.* 2022;3(5):100606.
4. Martin M. Cutadapt removes adapter sequences from high-throughput sequencing reads. *EMBnet journal.* 2011;17(1):10-12.
5. Dobin A, et al. STAR: ultrafast universal RNA-seq aligner. *Bioinformatics.* 2013;29(1):15-21.
6. Anders S, Pyl PT, Huber W. HTSeq-a Python framework to work with high-throughput sequencing data. *Bioinformatics.* 2015;31(2):166-169.

7. Frankish A, et al. GENCODE reference annotation for the human and mouse genomes. *Nucleic Acids Res.* 2019;47(D1):D766-D773.
8. Love MI, Huber W, Anders S. Moderated estimation of fold change and dispersion for RNA-seq data with DESeq2. *Genome Biol.* 2014;15(12):550.
